# Structural features of the protein kinase domain and targeted binding by small-molecule inhibitors

**DOI:** 10.1016/j.jbc.2022.102247

**Published:** 2022-07-10

**Authors:** Chris Arter, Luke Trask, Sarah Ward, Sharon Yeoh, Richard Bayliss

**Affiliations:** 1 Astbury Centre for Structural Molecular Biology, University of Leeds, Leeds, United Kingdom; 2 Faculty of Engineering and Physical Sciences, School of Chemistry, University of Leeds, Leeds, United Kingdom; 3 Faculty of Biological Sciences, School of Molecular and Cellular Biology, University of Leeds, Leeds, United Kingdom

**Keywords:** protein kinase, medicinal chemistry, structural biology, enzyme regulation, chemical biology, A-loop, activation loop, BBSRC, Biotechnology and Biological Sciences Research Council, CDK2, cyclin-dependent kinase 2, DFG, Asp-Phe-Gly, EGFR, epidermal growth factor receptor, ERK5, extracellular signal–regulated kinase 5, MEK, mitogen-activated protein kinase kinase, PDB, Protein Data Bank, PDK1, phosphoinositide-dependent protein kinase 1, PIF, PDK1-interacting fragment

## Abstract

Protein kinases are key components in cellular signaling pathways as they carry out the phosphorylation of proteins, primarily on Ser, Thr, and Tyr residues. The catalytic activity of protein kinases is regulated, and they can be thought of as molecular switches that are controlled through protein–protein interactions and post-translational modifications. Protein kinases exhibit diverse structural mechanisms of regulation and have been fascinating subjects for structural biologists from the first crystal structure of a protein kinase over 30 years ago, to recent insights into kinase assemblies enabled by the breakthroughs in cryo-EM. Protein kinases are high-priority targets for drug discovery in oncology and other disease settings, and kinase inhibitors have transformed the outcomes of specific groups of patients. Most kinase inhibitors are ATP competitive, deriving potency by occupying the deep hydrophobic pocket at the heart of the kinase domain. Selectivity of inhibitors depends on exploiting differences between the amino acids that line the ATP site and exploring the surrounding pockets that are present in inactive states of the kinase. More recently, allosteric pockets outside the ATP site are being targeted to achieve high selectivity and to overcome resistance to current therapeutics. Here, we review the key regulatory features of the protein kinase family, describe the different types of kinase inhibitors, and highlight examples where the understanding of kinase regulatory mechanisms has gone hand in hand with the development of inhibitors.

Deregulation of kinase function plays an important role in cancer as well as in immunological, inflammatory, neurodegenerative, metabolic, cardiovascular, and infectious diseases (1, 2, 3, 4, 5, 6, 7). This has led to protein kinases becoming one of the most important clinical targets and, as a result, the development of kinase inhibitors is a high priority in the pharmaceutical industry. However, for many years, kinases were thought of as difficult to target because of the inherent similarity of the ATP-binding site as well as the millimolar concentrations of ATP in the cell. In most cases, these challenges have been overcome, and a growing number of potent and relatively selective kinase inhibitors have been developed. There are many excellent reviews on different aspects of kinase inhibitors, providing an overview of trends, targets, and approaches (8, 9), novel targets and new technologies (2), and in-depth analysis of clinically approved kinase inhibitors (10). Most protein kinases have other domains that contribute to regulation of kinase activity, oligomerization state, or the recruitment of binding partners. For reasons of space, this review will focus primarily on the kinase domain itself. Here, we review the different types of protein kinase inhibitors in the context of the different structural and functional states of protein kinases and give examples to show how these features can be exploited in the development of kinase inhibitors.

The first crystal structure of a protein kinase, PKA, was published in a landmark article 30 years ago (11). This structure revealed a conserved structural core of protein kinases comprising an N-lobe that has a 5-stranded β-sheet (β1–β5) and at least one α-helix and a C-lobe that is mostly α-helical but with a small yet important β-sheet (β6–β7) (Fig. 1*A*). By convention, α-helices are named in alphabetical order starting at the N terminus of the structure, and β-strands are numbered starting from the N terminus of the protein with β1. However, in kinases, the convention is to name secondary structure elements based on the PKA structure, and for example, the conserved α-helix in the N-lobe of a protein kinase is usually called the αC-helix (or just C-helix), even in kinases that lack helices αA and/or αB. An ATP substrate molecule is sandwiched at the interface between the two lobes, and the surfaces of this cleft are formed from the β-sheets on both lobes (Fig. 1*B*). The phosphates of ATP are tucked under the Gly-rich loop that connects β1 and β2, interact with a conserved Lys residue on β3, and are connected to the C-lobe *via* a divalent cation, usually Mg^2+^. The lobes are connected by an ordered short linker sequence called the hinge that recognizes the adenine base of an ATP molecule through two H-bonds. The molecular interface between the two lobes is extensive and enriched in key structural and functional features. To the side of the ATP site, the two lobes come together through surfaces formed by the C-helix and the activation loop (A-loop), a dynamic feature that is a common site for phosphorylation that regulates kinase activity. The A-loop is also involved in recognizing protein substrates and forms part of a groove that extends along the front face of the C-lobe. However, in some kinases such as PINK1 (PTEN-induced kinase 1), the N-lobe is also involved in protein substrate recognition (12). The interface is completed by an extended loop between αC and β4 from the N-lobe, which packs against the β-sheet and helix αE from the C-lobe.

**Figure 1:**
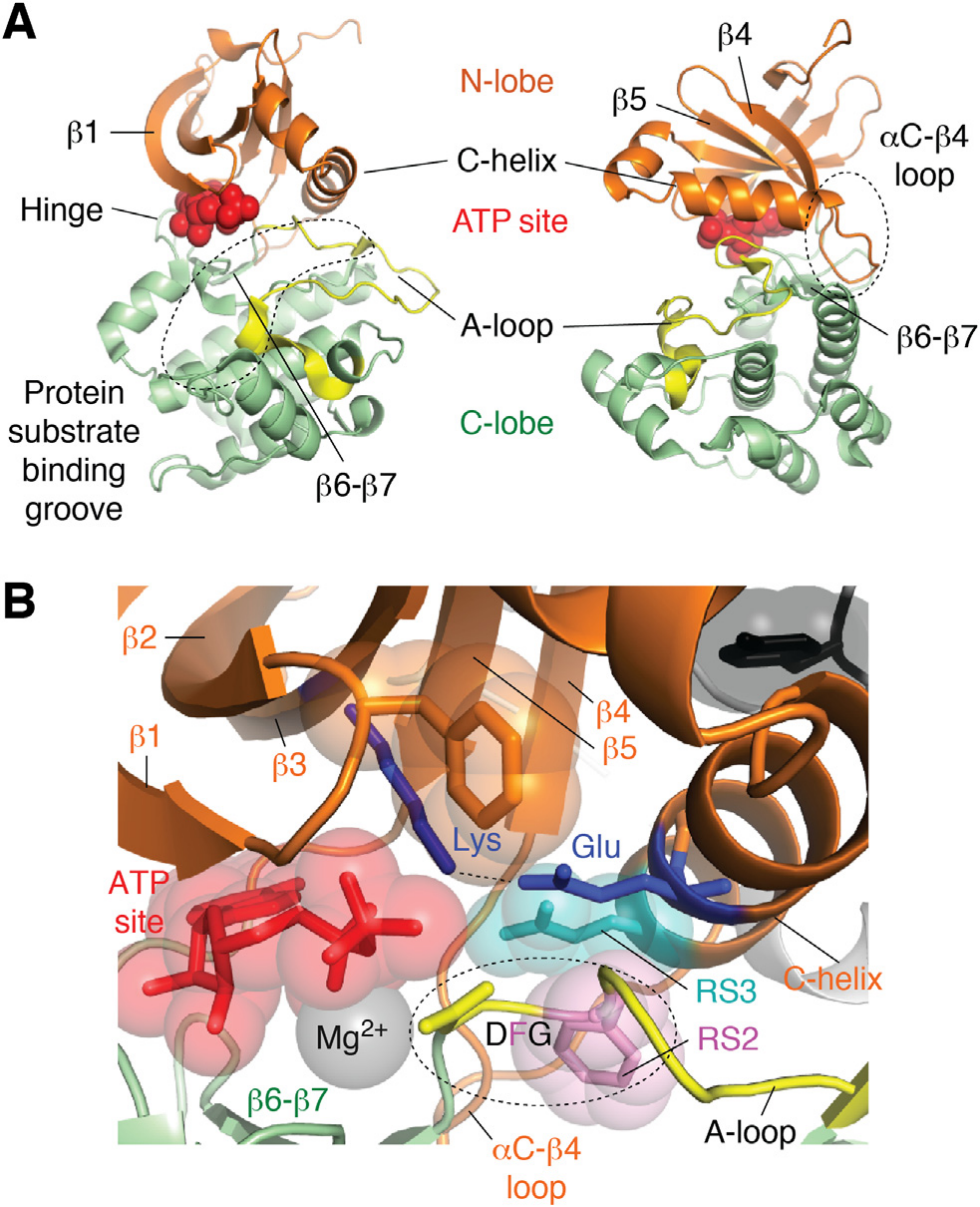
**Key structural features of a kinase domain.***A*, the domain comprises an N-terminal lobe (*orange*) and a C-terminal lobe (*green*) connected by a hinge sequence. The ATP-binding site (*red*) is at the interface of the two lobes. Adjacent to the ATP site, the interface between the two lobes is formed by the C-helix, a critical regulatory feature, and the A-loop, which forms part of the protein substrate-binding site. *B*, magnified view of the interface between the lobes, showing the critical interactions. A Lys residue on β3 and a Glu residue on the C-helix form a salt bridge that connects the ATP site to the C-helix. A crucial contact at the interface of the two lobes is formed by R-spine residue 3 (RS3), a side chain on the C-helix, and R-spine residue 2 (RS2). RS2 is also the central residue in a highly conserved 3-residue sequence, the DFG motif, located at the N terminus of the A-loop. Figures were generated using PyMOL. Images are based on the structure of Aurora-A kinase domain (Protein Data Bank entry: 1OL5) (109). A-loop, activation loop.

## The ins and outs of kinase regulation

**Figure 2:**
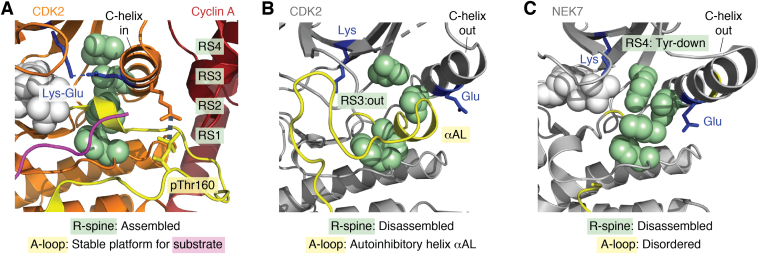
**Structures of kinases switching between an active C-helix in conformation and an inactive C-helix out conformation**. *A*, structure of active CDK2–cyclinA in complex with substrate (Protein Data Bank [PDB] entry: 1QMZ) (110). *B*, structure of inactive CDK2 (PDB entry: 1HCL) (111). *C*, structure of inactive NEK7 (PDB entry: 2WQN) (25). The side chains of the four R-spine residues are shown as *solid green spheres*.

Protein kinases act like molecular switches with a default “off” state, which is flipped to “on” in response to a signal. One regulatory mechanism is pseudosubstrate inhibition, in which a sequence from the kinase itself or another protein fills the protein substrate-binding site. For example, PKA is inhibited by a 20 amino acid peptide region from the protein kinase inhibitor protein (13, 14). Kinases may also be inactive because the key active site residues are displaced from their proper positions. Protein kinases that are primed for catalysis exhibit a specific active conformation in which the catalytic and regulatory residues are aligned (Fig. 2*A*). One hallmark of the active conformation is a stacked configuration of four side chains in the hydrophobic core of the kinase domain, called the regulatory R-spine, labeled RS1–RS4 (Fig. 2*A*) (15, 16). There is another hydrophobic spine, the catalytic (or C-spine), that passes through the adenine ring of the ATP/ADP substrate. Together, the two spines form a hydrophobic core connection between the two kinase lobes. Another hallmark is the stabilization of the A-loop through phosphorylation and/or protein–protein interactions into a platform to support the binding of protein substrates (17, 18). In contrast, crystal structures of protein kinases captured in the “off” state show a diverse range of conformations, but a common theme is the disruption of interactions involving the C-helix and the A-loop.

The C-helix is a critical structural element in the allosteric behavior of protein kinases (18, 19). The C-helix is involved in three interactions that are hallmarks of kinase activity: it contributes one hydrophobic amino acid side chain to the R-spine (RS3); it has a conserved Glu that, in the active kinase, forms a salt bridge with a Lys on the β3 strand; it interacts with the A-loop, often through a salt bridge with the activating phosphate group. These interactions are present in the structures of active kinases in which the C-helix is tightly packed against the rest of the kinase domain, termed C-helix in or C-in (Fig. 2*A*). Inactive states of some kinases are characterized by a looser attachment of the C-helix, often called C-helix out or C-out (Fig. 2, *B* and *C*). Many kinases are regulated by a C-helix out/C-helix in mechanism, including the well-studied tyrosine kinases Src and epidermal growth factor receptor (EGFR) (20). Lacking the hallmark interactions, the C-helix may be more dynamic as is apparent in crystal structures of kinases such as NEK2 (NIMA related kinase 2), in which the electron density in the vicinity of the C-helix is poor relative to the rest of the kinase domain (21). The C-helix out position is stabilized in some kinases through autoinhibitory structural features that must be released as the first step in kinase activation. For example, C-helix out in inactive CDK2 (cyclin-dependent kinase 2) is stabilized by a refolded conformation of the A-loop that occupies the space between the Lys and Glu (Fig. 2*B*) (22). Binding of cyclinA brings about a substantial conformational change: the A-loop is displaced from the active site and is trapped under the C-helix, which moves “in.” Phosphorylation of the A-loop on Thr160 reinforces this change because an additional salt bridge is formed between Arg50 of the C-helix and the phosphorylated Thr160 on the A-loop (23). Mutations in the A-loop that cause constitutive kinase activity occur frequently in cancers, and these might act to destabilize autoinhibitory conformations and/or stablize an active conformation (24).

An alternative mechanism of autoinhibition through C-out stabilization is observed in kinases such as NEK7 and IRE1 that have a Tyr residue at the RS4 position (Fig. 2*C*) (25, 26). Here, the Tyr side chain is oriented to fill the space created by the RS3 residue on the C-helix having an out position, and this conformation is further stabilized by a H-bond formed between the Tyr side chain and the A-loop. Dimerization of the NEK7 kinase domain induces a conformational change in the kinase, releasing the Tyr side chain and enabling autophosphorylation (27). In common with many kinases, the dimeric interface in NEK7 is centered on the loop that connects the C-helix to the β4 strand (Fig. 1*A*). The αC–β4 loop is usually 8 amino acids long, with a conserved sequence motif “Φ-X-H-X-N-Φ-Φ-X“ (where Φ is hydrophobic and X is any amino acid) but can be much longer (*e.g.*, over 30 amino acids in PINK1 from *Tribolium castaneum*; Protein Data Bank [PDB] entry: 5OAT) (28). It is a highly ordered structure that serves to clamp together the C-helix and β4 through the interactions between two R-spine residues (RS3 and RS4). The loop also forms extensive interactions with the C-lobe (E-helix and β7 strand). The inactive state of RTKs can be stabilized by an additional set of interactions within the loop called the “molecular brake,” first discovered in the fibroblast growth factor receptor family (29). The αC–β4 loop is a hot spot for disease-associated mutations that promote kinase activity through disruption of the molecular brake (30). The αC–β4 loop is also a hot spot for regulatory protein–protein interactions, forming the dimer interface that governs the activation of many kinases, including NEK7 and BRAF.

**Figure 3:**
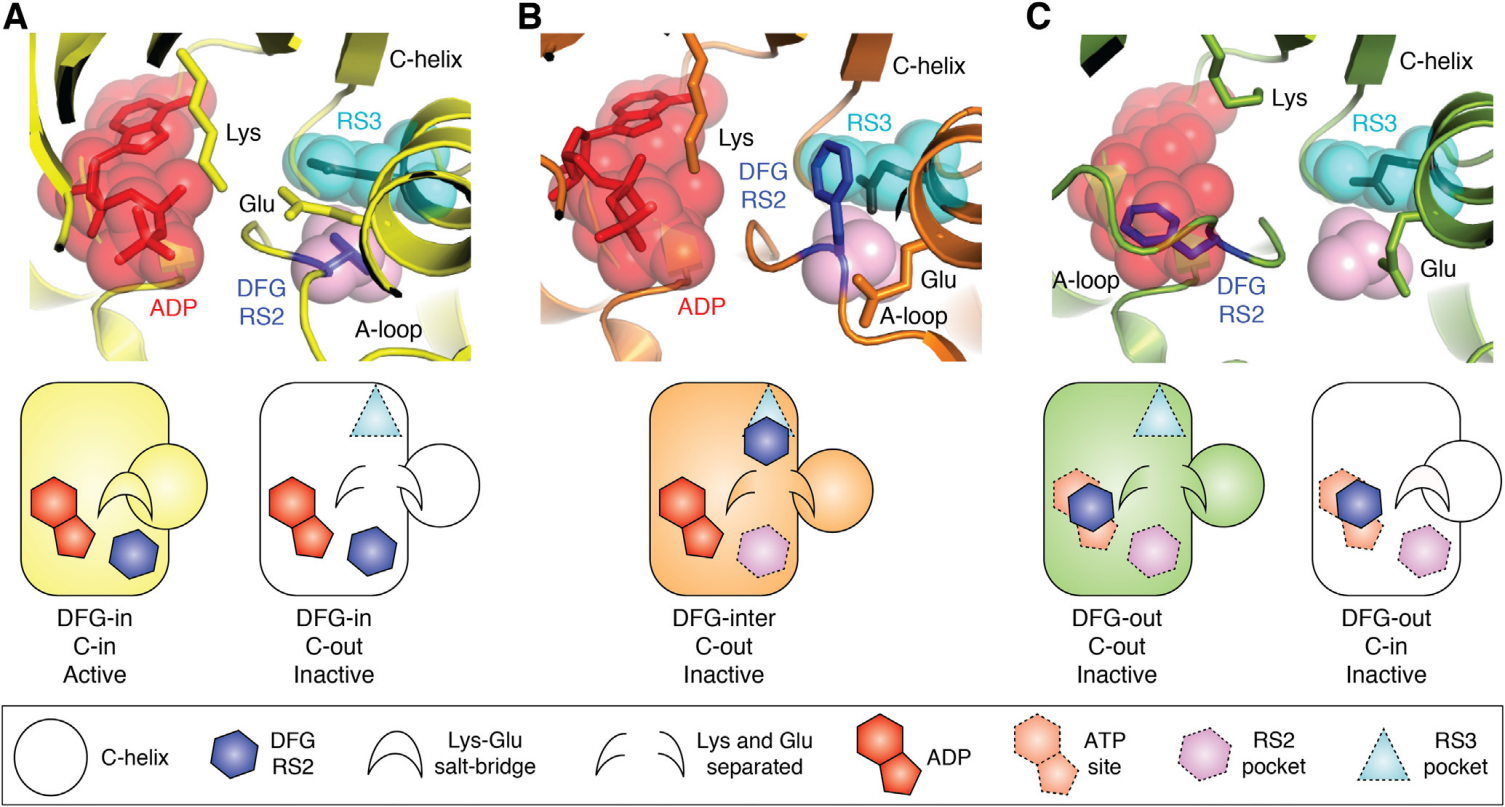
**Three positions of the DFG motif and two allosteric pockets adjacent to the ATP site.** Crystal structures of Aurora-A in three different DFG conformations, in which the Phe side chain (RS2) is highlighted in a *blue* color. *A*, DFG-in (Protein Data Bank [PDB] entry: 1OL5) (109). *B*, DFG-inter (PDB entry: 5L8L) (33). *C*, DFG-out (PDB entry: 6HJK) (112). Three regions are shown as *translucent spheres*, marked in all three panels using the positions of atoms from *A*: ATP site—*red*; RS3/back pocket—*cyan*; and RS2/front pocket—*pink*. Other notable features are labeled on the crystal structures. Schematic representations of the crystal structures highlight key features such as the presence or the absence of allosteric pockets, and are color coded as the crystal structures, or presented in *white* for additional conformations that are not shown as crystal structures.

A tripeptide motif with the consensus Asp-Phe-Gly (DFG) at the N-terminal end of the A-loop acts as a pivot point for the conformation of the A-loop (Fig. 3). The two most common conformations of the DFG motif are DFG-in, in which the A-loop interacts with the C-helix (Fig. 3*A*), and DFG-out, in which the A-loop is directed away from the C-helix (Fig. 3*C*). The active conformation of kinases is always DFG-in, C-in, and the central residue of the DFG motif (the RS2 residue) packs against the RS3 residue from the C-helix to form the central interaction of the R-spine. In contrast, the RS2 and RS3 residues are separated in the inactive DFG-out conformation of kinases. This simple classification of DFG conformations was extended in a recent study (31). Analysis of the main-chain geometry of the Asp–Phe dipeptide and the preceding residue, and the rotamer of the Phe side chain, identified eight distinct conformations comprising six subclasses of DFG-in, one DFG-out, and a third, called DFG-inter (Fig. 3*B*). We and others previously referred to DFG-inter as DFG-up, but we agree that intermediate is a clearer description of this conformation (32, 33, 34). DFG-inter is only found in kinase structures that also have the C-out conformation because the Phe side chain fits into the space that is occupied by the RS3 residue in the C-in conformation (Fig. 3*B*). In contrast, DFG-in and DFG-out conformations may be present in kinase structures that are either C-in or C-out. Of the six subclasses of DFG-in conformations, one is found in kinases primed for catalysis, and the others reflect inactive states of the kinase. The active DFG-in conformation has the Asp side chain pointing into the ATP-binding site and interacting with a magnesium ion that bridges between the protein and the phosphates, and the Phe side chain pointing into the hydrophobic core of the kinase structure at the interface between the N- and C-lobes, whereas these features are inconsistent in other subclasses of DFG-in conformation. This study also found a correlation between the conformation of the DFG motif and the position of the C-helix: 95% of kinase structures with the active DFG-in conformation also have the C-in position (*yellow*, Fig. 3*A*); 77% of kinase structures in a common inactive DFG-in conformation have the C-helix out (*white*, Fig. 3*A*) (31). There are excellent online resources available to explore the conformational diversity of protein kinases and kinase–inhibitor complexes, notably KINCORE and KLIFS (35, 36).

The classification of different DFG-in conformations provides the impetus for structural and mechanistic studies to further our understanding of kinase regulatory mechanisms. Knowledge of the different conformations of the DFG motif and how this affects the geometry of the ATP site is an important consideration in the design of kinase inhibitors. Displacement of either the C-helix or DFG motif from the “in” position breaks their interaction and opens up pockets in and around the ATP-binding site that can be exploited in drug discovery (37, 38). These features are usually named interchangeably as an allosteric pocket, back pocket, or similar. For the purposes of relating kinase inhibitor design to kinase regulatory mechanisms, it would be helpful to more clearly define which region of the kinase structure is being described. Here, we will use descriptions based on the key residue that occupies that pocket in the active conformation of the kinase (Fig. 3). The RS3 pocket is at the C-terminal end of the C-helix, is occupied by the RS3 residue in an active kinase structure, and is vacated in a C-out conformation. The RS2 pocket is underneath the C-helix, is occupied by the RS2 residue (also known as the Phe of the DFG motif) in active kinase structures, and is vacated in DFG-out and DFG-inter conformations. These pockets are flexible, and typically, an inhibitor will bind to an expanded form of the pocket, and some will occupy more than one pocket.

Most of the structural understanding of kinase regulation has been obtained using X-ray crystallography, starting from the first structure of PKA in 1991 (11). More recently, spectroscopic methods have generated information on the conformations adopted by kinases in solution and the interconversions between them. For example, the transitions of the Aurora-A A-loop between different states has been monitored using single-molecule approaches (39). In a recent tour de force, the structure of the tyrosine kinase domain of ABL1 was analyzed using NMR spectroscopy, generating a wealth of information that augments the many previous studies on this protein (40). In solution, unliganded ABL1 exists primarily in an active-like conformation with the DFG motif and C-helix both “in.” Minor populations of ABL1 molecules in two distinct inactive states were also observed. One of the inactive states is a DFG-out C-in conformation that is very similar to the crystal structure of ABL1 in complex with the inhibitor PD173955. The other inactive state is a DFG-out, C-out conformation that resembles the crystal structure of ABL1 in complex with the inhibitor imatinib, although there are some differences, for example, in the disposition of the Gly-rich loop and C-helix. The authors conclude that the presence of pre-existing conformations of the kinase that closely resemble the inhibitor-bound state may favor high-affinity binding.

## In and around the ATP site: Type I and type II inhibitors

There are more than 90 clinically approved drugs that target protein kinases https://www.ppu.mrc.ac.uk/list-clinically-approved-kinase-inhibitors, of which at least 63 have been approved by the Food and Drug Administration (10). The vast majority of these compounds compete with ATP for inhibition. The structural similarity of the ATP site has allowed the identification of common hinge-binding scaffolds (41) and enabled the prediction of binding modes and key interactions of novel ligands providing a rationale for inhibitor design (42). Approaches for developing ATP-competitive inhibitors are therefore relatively mature and can be used to rapidly generate libraries of compounds for inhibitor hit discovery (43). In one study, high-throughput kinase profiling utilizing a library comprising two distinct chemical scaffolds identified potent inhibitors against several kinases that enabled the efficient development of a multitude of selective kinase inhibitors (44): TNK2 (tyrosine kinase ACK1) (45), PI3K-δ/γ (46), the Aurora kinases (47, 48), LRRK2 (leucine-rich repeat kinase 2) (49), extracellular signal–regulated kinase 5 (ERK5) (50), and DCLK1 (51, 52). By utilizing kinase profiling, inhibitors targeting a few kinases can be optimized into highly selective inhibitors of those distinct kinases. This was the case for XMD8-92, which was developed into selective ERK5 inhibitor AX15836 (50). Aided by cocrystal structures, XMD8-92 was also developed into DCLK1-IN-1, a selective DCLK1 inhibitor, by exploiting the differences between ERK5 (leucine) and DCLK1 (methionine) in the gatekeeper residue that sits at the back of the ATP site (51). The gatekeeper residue is a frequent site of point mutations in kinases that confer resistance, and therefore, it is a logical strategy to use this property to fine-tune kinase inhibitor selectivity. DCLK1 has been identified as an understudied druggable kinase whose overexpression is linked to multiple human cancers such as colorectal and gastric cancer as well as pancreatic ductal carcinoma. The selective compound DCLK1-IN-1 helped to establish cell motility as a role of DCLK1 in patient-derived pancreatic ductal carcinoma organoids and differentiate results obtained with earlier and less selective probes (53, 54).

**Figure 4:**
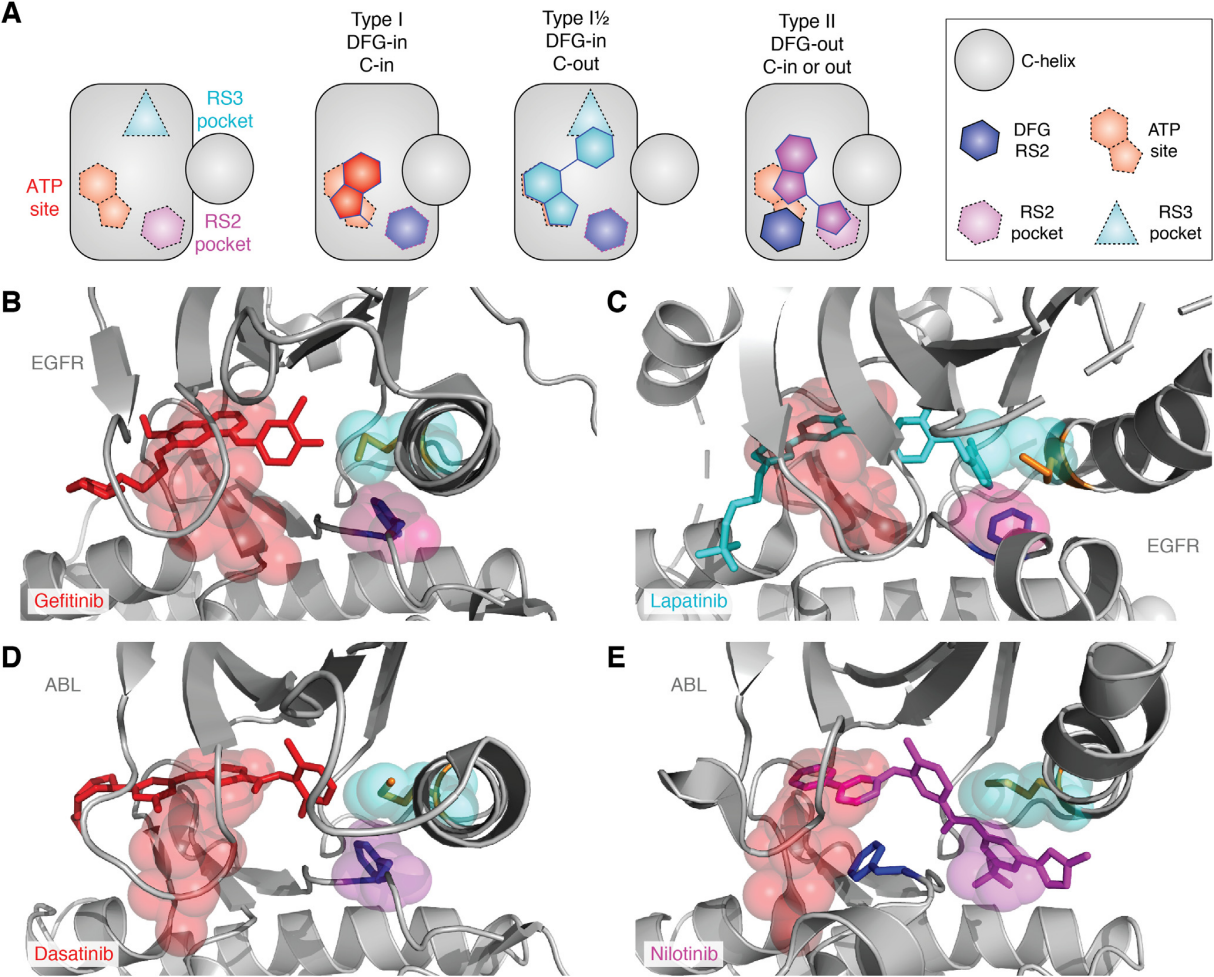
**Different types of ATP-competitive kinase inhibitors.***A*, schematic illustrations of the two allosteric pockets adjacent to the ATP site and how they are exploited by different kinase inhibitors. *B*, crystal structure of type I inhibitor gefitinib (*red*) bound to EGFR (*gray*) (Protein Data Bank [PDB] entry: 2ITY) (113). *C*, crystal structure of type I½ inhibitor lapatinib (*cyan*) bound to EGFR (*gray*) (PDB entry: 1XKK) (114). In *B* and *C*, the *translucent spheres* mark the positions of the ATP site (*red*), RS2 pocket (*magenta*), and the RS3 pocket (*cyan*) in EGFR (PDB entry: 2ITX) (113). Note that RS2 and RS3 pockets are occupied by the RS2 and RS3 residues, respectively, in the active EGFR structure shown in *B*, and the RS3 pocket is opened in the C-out conformation of inactive EGFR shown in *C*, and is occupied by the type I½ inhibitor. *D*, crystal structure of type I inhibitor dasatinib (*red*) bound to ABL1 (*gray*) (PDB entry: 2GQG) (115). *E*, crystal structure of type II inhibitor nilotinib (*magenta*) bound to ABL1 (*gray*) (PDB entry: 3CS9) (116). *D* and *E*, the *translucent spheres* mark the positions of the ATP site (*red*), RS2 pocket (*magenta*), and the RS3 pocket (*cyan*) in ABL1 (PDB entry: 2G2I) (117). Note that RS2 and RS3 pockets are occupied by the RS2 and RS3 residues, respectively, in the active ABL1 structure shown in *D*, and the RS2 pocket is opened in the DFG-out conformation of inactive ABL1 shown in *E*, and is occupied by the type II inhibitor. EGFR, epidermal growth factor receptor.

ATP-competitive kinase inhibitors are usually classified into three “types,” all of which mimic ATP and make interactions with the hinge region within the kinase ATP site but vary in the conformational state of the kinase they interact with, and the additional interactions they make (Fig. 4) (37, 38). Type I inhibitors are defined as small molecules that bind to the active conformation of the kinase (DFG-in and C-in; Fig. 4, *B* and *D*); type I½ inhibitors bind to the inactive DFG-in, C-out conformation of the kinase (Fig. 4*C*); type II inhibitors bind to the inactive DFG-out conformation of the kinase (C-in or C-out; Fig. 4*E*). While type I inhibitors are mostly confined to the ATP site, type I½ and type II inhibitors extend into the distinctive pockets that are opened up in the specific inactive conformations they bind: Type I½ compounds occupy the RS3 pocket that is vacated by the RS3 residue when the C-helix is out; type II compounds occupy the RS2 pocket that is vacated by the Phe side chain of the DFG motif when it is flipped out (Fig. 4, *A* and *E*). These generalized definitions are helpful because they connect the design and development of inhibitors to the key regulatory mechanisms of kinases. There are, however, additional levels of complexity and, to an extent, each kinase presents a different set of challenges for the development of inhibitors.

The Ser/Thr kinase BRAF provides an instructive example of how different designs of inhibitors can produce distinct biological outcomes, with important consequences for their application in cancer therapy, reviewed by Ref. (55). RAF kinases (ARAF, BRAF, and CRAF) are components of the oncogenic RAS–mitogen-activated protein kinase pathway that are activated by RAS-GTP and that propagate signaling by phosphorylating another kinase, mitogen-activated protein kinase kinase (MEK). RAF kinases may be activated through homodimerization, heterodimerization with each other, or heterodimerization with the pseudokinase KSR (kinase suppressor of RAS), and these events may be facilitated by dimeric 14-3-3 proteins (56). BRAF is the most thoroughly investigated family member owing to the prevalence of mutations in several cancer types, including melanoma and lung cancer (57). Oncogenic mutations in BRAF drive constitutive kinase activity through single amino acid changes (notably the A-loop mutation V600E in melanoma), deletions within regulatory regions (*e.g.*, β3–αC), or fusion to another protein such as the calcium-activated chloride channel TTYH3 (58, 59, 60, 61).

The first clinically approved BRAF inhibitors, vemurafenib and dabrafenib, are effective in melanoma driven by V600E BRAF, but they are less effective in other contexts and can accelerate the progression of cutaneous squamous cell carcinomas (55). This is because, in cells with upregulated RAS activity through increased signaling or RAS mutation, these inhibitors induce activation of BRAF or CRAF (62). This is consistent with previous observations of enhanced signaling by BRAF harboring mutations that stabilize the C-spine by blocking the ATP site (15). A molecule of BRAF may therefore act as an allosteric activator of another molecule of BRAF (or CRAF). One way to overcome this issue is to ensure that both BRAF protomers in the dimer are occupied by inhibitor. Indeed, type II BRAF inhibitors, such as LY3009120, are equally able to inhibit monomeric and dimeric BRAF (63). A recent screen of kinase inhibitors against the dimeric form of BRAF identified ponatinib, the ABL1 inhibitor, as type II inhibitor of BRAF that extends into an additional allosteric pocket underneath the C-helix (64). Structure-guided design based on ponatinib/BRAF yielded the compound PHI1, which showed positive allostery, meaning that binding of an inhibitor to the second protomer in a dimer is preferred. The development of BRAF inhibitors has implications for the design of inhibitors that target other kinases that are activated through dimerization, as different types of inhibitors may result in different downstream effects.

**Figure 5:**
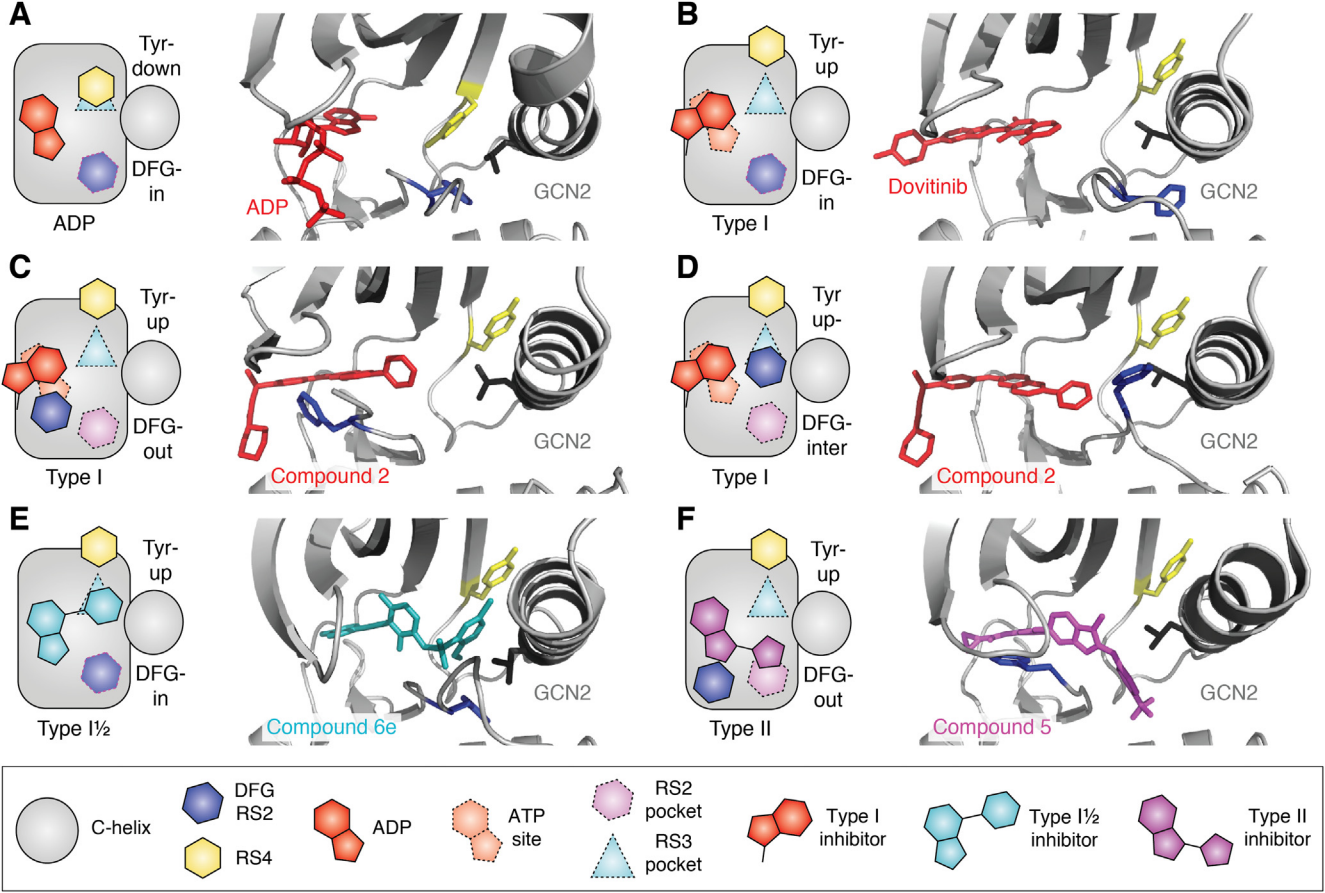
**Type I, I½, and II inhibitors of GCN2.** The schematic illustration of each GCN2–ligand complex has shapes with *dashed edges* that indicate pockets (*red*—ATP site; *cyan*—RS3 pocket; and *magenta*—RS2 pocket) and shapes with *solid edges* indicate molecular features that occupy pockets (*red*—ADP or type I inhibitor; *cyan*—type I½ inhibitor; *magenta*—type II inhibitor; *blue*—DFG RS2; and *yellow*—RS4). *A*, yeast GCN2 (Protein Data Bank [PDB] entry: 1ZYD) (65). *B*, human GCN2–dovitinib complex (PDB entry: 7QQ6) (34). *C*, human GCN2–compound 2 complex in DFG-out conformation (PDB entry: 7QWK) (34). *D*, human GCN2–compound 2 complex in DFG-inter conformation (PDB entry: 7QWK) (34). *E*, human GCN2–type I½ complex (PDB entry: 6N3O) (66). *F*, human GCN2–type II complex (PDB entry: 6N3L) (66).

To add further levels of complexity to the consideration of designing inhibitors, there is great diversity in the structures of inactive kinase states, and the mechanisms through which dimerization contributes to activation are varied. It is therefore not always clear which pockets are available for targeting, and what the outcome might be. GCN2 is one of four Ser/Thr kinases within the integrated stress response pathway, and it specifically regulates amino acid homeostasis in response to nutrient starvation (4). It is activated through binding to uncharged tRNA and phosphorylates the eukaryotic initiation factor 2 alpha on Ser51, reducing global protein translation. The crystal structure of inactive yeast GCN2 kinase domain revealed a dimer formed through interactions between the N-lobes, centered on the αC-helix and β4 strand (65). More recently, structures of the human GCN2 kinase domain revealed an alternative dimeric conformation, with an interface that extends over the αC–β4 loop, which more closely resembles the active dimeric configuration of other integrated stress response kinases such as PKR (34, 66). None of the GCN2 structures reported to date represents an active kinase because the C-helix is out and the Lys–Glu salt bridge is broken, but there are parallels with other kinases that suggest how a change in dimerization interface may be coupled to kinase activation. In the yeast GCN2 structure, the Tyr side chain of RS4 points inward, filling the RS3 pocket left vacant by the out position of the C-helix (Fig. 5*A*). A similar conformation of RS4 was previously observed in NEK7 and IRE1 and was established as an autoinhibitory feature in these kinases (26, 27). The PKR-like dimer in human GCN2 structures also has the C-helix out, but the RS4 residue does not fill the RS3 pocket and instead sits on the surface of the protein, as would be expected in an active kinase (Fig. 5, *B*–*F*). The change in position of the RS4 residue is consistent with release of autoinhibition, as was observed in NEK7 and IRE1, but it is not known if the RS4 residue has an autoinhibitory function in GCN2. Human GCN2 has sufficient flexibility to adopt a range of conformations. Indeed, in complex with a type I inhibitor, an aminoquinazoline, human GCN2 was captured in both DFG-out and DFG-inter conformations within the same crystal (Fig. 5, *C* and *D*) (34). Type I½ inhibitors of GCN2 have also been reported, based on a series of sulfonamide compounds that were first identified as BRAF inhibitors (66). In one example, compound 6e, the sulfonamide is capped with a disubstituted pyridine that fits into the open RS3 pocket of the human GCN2 protein (Fig. 5*E*). Another series of compounds developed for BRAF provided an example of a GCN2 inhibitor with a type II–binding mode, compound 5, in which a phenyl group substituted with trifluoromethyl fits into the RS2 pocket that is opened by the DFG-out conformation of the A-loop (Fig. 5*F*). The C-out conformation of GCN2 and the flexibility of its A-loop thus provide opportunities for the development of type I½ or type II inhibitors. This might not have been expected, based on the yeast GCN2 structure, and this highlights broader questions for the field. Given the flexibility of kinases, can the range of conformations that the kinase may potentially adopt be rigorously characterized or modeled? Can we then predetermine a suitable strategy for each kinase—whether type I, I½, or II? Achieving the required selectivity for a chemical probe with ATP-competitive inhibitors remains a challenge, and there is an increasing effort to target kinases with allosteric inhibitors.

## Beyond the hinge: Type III inhibitors

Inhibitors that do not compete with ATP provide the opportunity to gain greater selectivity over other kinases by binding to allosteric sites that are less conserved than the ATP-binding site (67). Allosteric inhibitors that bind adjacent to the ATP site are classified as type III and those that bind to other more distant sites as type IV. Type III inhibitors of MEK1/2 (trametinib, cobimetinib, and binimetinib) are approved as cancer therapeutics, used in the treatment of melanoma and non–small cell lung cancer patients who have mutations in BRAF, in combination with a BRAF inhibitor. Another MEK1/2 type III inhibitor (selumetinib) is approved for treatment of neurofibromatosis type 1 (10).

Several approaches have been followed to discover allosteric MEK inhibitors. A screen against a MEK1–mitogen-activated protein kinase cascade assay identified the compounds PD098059 and PD184352 that inhibited MEK1 activation (68, 69, 70). Another allosteric inhibitor of MEK (JTP-70902) was identified from a phenotypic screen as an inducer of p15^INK4b^ in HT-29 human colon cancer cells (71). Other early examples of type III inhibitor discovery include MK-2206, an AKT inhibitor identified through a high-throughput kinase activity assay, as well as PD0325901, developed from CI-1040, a MEK inhibitor identified in a high-throughput cell-based assay (72, 73). One example of a type III inhibitor identified through a targeted approach is JBJ-04-125-02, an EGFR inhibitor developed from EAI045 (74, 75). The targeted approach involved high-throughput screening of compounds against a clinically relevant EGFR mutant in the presence of 1 μM ATP, with active compounds subsequently screened at a higher ATP concentration to eliminate ATP-competitive inhibitors (75). Kinase inhibitors can only be assigned unambiguously as type III through further biophysical studies, such as determination of a cocrystal structure with the target kinase.

**Figure 6:**
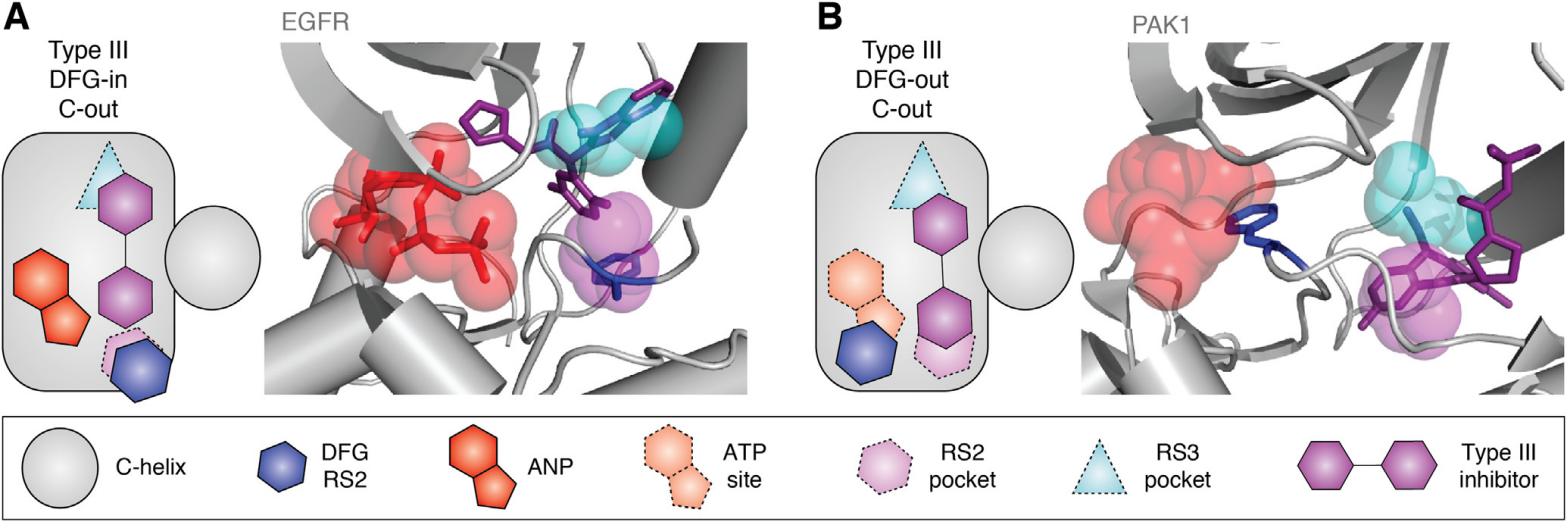
**Type III inhibitors bind to pockets adjacent to the ATP site.***A*, the type III inhibitor EAI045 (*purple*) binds to EGFR (*gray*) in a DFG-in, C-out conformation (Protein Data Bank [PDB] entry: 6P1L) (78). A nonhydrolyzable ATP analog (ANP) is bound to the ATP site (*red*). The positions of the RS2 pocket (*magenta*) and RS3 pocket (*cyan*) are superposed from an ATP analog–bound crystal structure of EGFR (PDB entry: 2GS6) (118). *B*, the type III inhibitor dibenzodiazepine compound 2 (*purple*) binds PAK1 (*gray*) in a DFG-out, C-out conformation (based on PDB entry: 4ZJJ) (80). The positions of ATP site (*red*), RS2 pocket (*magenta*), and RS3 pocket (*cyan*) are superposed from an ATP-bound crystal structure of PAK1 (PDB entry: 3Q53) (119). EGFR, epidermal growth factor receptor.

Type III inhibitors typically bind to the DFG-in, C-out conformation of the kinase, occupy the RS3 pocket, and interact with the Phe side chain of the DFG motif (Fig. 6*A*). Examples of compounds with this binding mode include the archetypal MEK1 inhibitors such as PD318088 (related to PD184352) and trametinib (related to JTP-70902) as well as type III EGFR inhibitors such as EAI045 (76, 77, 78). As such, there is an overlap between the binding mode of type III inhibitors and type I½ inhibitors, both of which occupy the RS3 pocket. Type III compounds form additional interactions with the C-helix and occupy the space between the separated Lys and Glu salt-bridge residues. They may also contact the ATP molecule. Interestingly, the conformation of EGFR and MEK bound to type III inhibitors falls into the most common subgroup of inactive DFG-in conformations (31). It therefore seems likely that type III inhibitors could be developed to target this specific conformation in many other kinases, perhaps inspired by the lessons learned from MEK and EGFR (79).

Some type III inhibitors bind to the DFG-out, C-out conformation (Fig. 6*B*). These include a series of PAK1 inhibitors that were identified in a fragment-based screen and characterized as allosteric site binders by X-ray crystallography (80). The dibenzodiazepine scaffold of these compounds occupies the RS2 pocket, and PAK1 selectivity and potency were optimized with the addition of a small hydrophobic group that occupies the RS3 pocket, and a bulky amine group that fills the space between the separated Lys and Glu salt-bridge residues. These PAK1 inhibitors exploit the large cavity that includes the RS2 and RS3 pockets in the DFG-out, C-out conformation of this kinase. These pockets are not always available in the DFG-out, C-out conformation, as exemplified by allosteric inhibitors of AKT such as miransertib (ARQ 092) (81, 82). This inhibitor binds to a highly distorted inactive conformation of AKT characterized by an extreme “out” position of the C-helix, which may be disordered. A Pleckstrin homology domain that is N-terminal to the kinase domain of AKT occupies the space vacated by the C-helix and thus occupies the RS3 pocket. The type III AKT inhibitor sits at the interface of the kinase and Pleckstrin homology domains, occupying the RS2 pocket. The cocrystal structure of the allosteric MK-2206 inhibitor bound to AKT is not available in the PDB, but modeling studies suggest that it may also have a type III binding mode (83). The PAK1 and AKT inhibitors take advantage of regulatory mechanisms specific to these kinases, a logical strategy for the development of potent and selective compounds.

## Outside the ATP site: Type IV inhibitors

**Figure 7:**
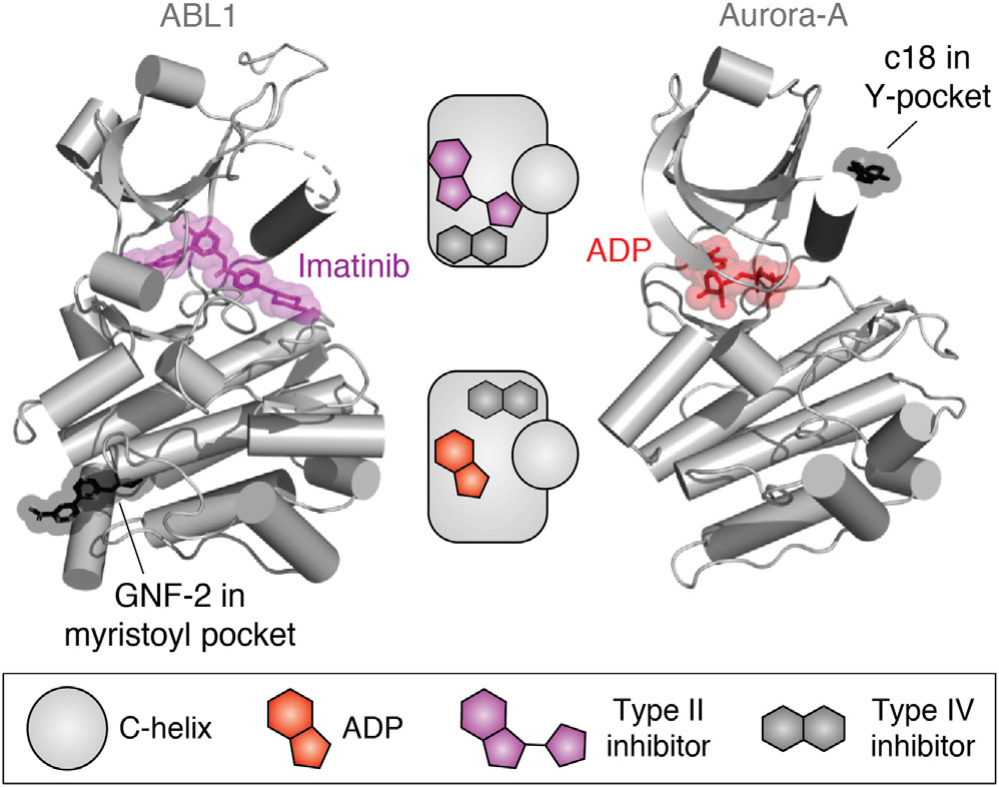
T**ype IV inhibitors bind to allosteric pockets distant from the ATP site.***Left*, crystal structure of ABL1 in complex with imatinib (*magenta*) and GNF-2 (*black*) (Protein Data Bank [PDB] entry: 3K5V) (89). *Right*, crystal structure of Aurora-A in complex with ADP (*red*) and compound 18 (*black*) (PDB entry: 5OS5) (96). Note that the Y-pocket of Aurora-A is structurally equivalent to the PIF pocket of AGC kinases such as PDK1. PDK1, phosphoinositide-dependent protein kinase 1; PIF, PDK1-interacting fragment.

Type IV kinase inhibitors are defined as kinase inhibitors that bind to an allosteric site on the kinase catalytic domain, remote from the ATP site (84). Allosteric sites are shallower, broader, more solvent exposed, and less well defined than the ATP-binding site. Targeting allosteric sites is less straightforward but provides opportunities for imparting enhanced selectivity. This approach can be exemplified by the development of asciminib (ABL001), the first type IV allosteric inhibitor of BCR-ABL1 to enter clinical trials (85). Asciminib was recently granted Food and Drug Administration approval for treatment of chronic myelogenous leukemia in adult patients with Philadelphia chromosome–positive chronic myelogenous leukemia in chronic phase who have been previously treated with two or more kinase inhibitors or who have T315I mutation. Asciminib was developed from GNF-2 and GNF-5, the first well-characterized type IV inhibitors of BCR-ABL1, shown to perturb substrate binding through an allosteric mechanism upon compound binding to the C-terminal myristate pocket of ABL1 kinase (Fig. 7) (86). Binding of GNF-2 and GNF-5 to the myristate site is characterized by a trifluoromethoxy group bound deep in a hydrophobic pocket, with the amide extending out of the myristate pocket toward the surface of the protein. Crucial water-mediated hydrogen bonding interactions between the pyrimidine and Tyr454, and the amine with Ala452, confer exquisite selectivity to the GNF compound series for ABL1 over similar myristate-binding proteins (87). Binding of GNF-2 and GNF-5 to ABL1 containing SH2 and SH3 domains removes the kinase activity through a structural change, “bending” a C-terminal helix local to the myristate site, allowing the SH2 and SH3 domains to dock against the kinase domain, thereby mimicking the natural autoinhibition mechanism lost in the BCR-ABL1 fusion protein, as characterized with an NMR-based biophysical assay (88). Despite having good potency and selectivity against WT BCR-ABL1, GNF-2 and GNF-5 displayed reduced efficacy against certain BCR-ABL1 mutants, with the gatekeeper residue T315I mutation displaying a significant reduction in potency (IC_50_ >10 μM). However, when GNF-5 was combined with nilotininb, a type II ABL1 inhibitor, the combined effect was to rescue the inhibitory potency of both compounds, suggesting combinatorial therapy of both allosteric and orthosteric binders at the myristate- and ATP-binding site, respectively, may hold potential for the treatment for cancers displaying this T315I mutation, commonly found in treatment-resistant cells (89).

Using the NMR-based conformational assay developed and utilized in the discovery of GNF-2 and GNF-5, a fragment screen was performed to identify hit fragments that were then elaborated to generate early structure–activity relationships, followed by thorough structure-based design to result in asciminib (85). While asciminib binds to the same myristate pocket and in a similar manner to GNF-2 and GNF-5, it retains inhibition against all ATP-site mutations of BCR-ABL1, including the T315I mutation, a major advancement from the previous compounds. Like GNF-5, the combination of asciminib with ATP-competitive BCR-ABL1 inhibitors has been shown to overcome acquired resistance mutations from either of the compounds in isolation (90). It is the orthogonal mode of inhibition between asciminib and ATP-competitive inhibitors that has led to asciminib being evaluated in >15 clinical trials as a single treatment agent and in combination with ATP-site competitors, highlighting the potential of type IV inhibitors.

Other kinases have different allosteric pockets that can be targeted with type IV inhibitors. For example, phosphoinositide-dependent protein kinase 1 (PDK1) is a major regulator of the AGC family of kinases, with at least 23 downstream kinases dependent on activation by PDK1 (91). Distant to the ATP-binding site, the PDK1-interacting fragment (PIF) pocket is crucial for not only recruitment of these downstream substrate kinases but also stimulating the kinase activity of PDK1 itself presenting this site an attractive target for development of type IV allosteric inhibitors (92, 93). The PIF pocket lies on the N-lobe of the kinase domain, between the C-helix and the β4 strand, a hot spot for kinase regulation *via* protein–protein interactions that may present a broader opportunity for targeting kinase activity (19). *In silico* docking, conventional medicinal chemistry efforts, and high-throughput crystallography have been used to discover inhibitors that target the equivalent hydrophobic pockets in PKC (94) and Aurora-A (95, 96), respectively (Fig. 7). Allosteric communication between the ATP site and the PIF pocket is bidirectional: ATP-competitive inhibitors of PDK1 and Aurora-A modulate the binding of protein partners (97, 98). Although many of the tools to develop type IV inhibitors exist, progress will be relatively slow because the design of these inhibitors is still exploratory, whereas we only have a few examples and lack a solid theory of design. The development of type IV inhibitors is also limited by our understanding of regulatory mechanisms, which are incomplete even in some of the most highly studied kinases. Indeed, our knowledge of kinase regulation often progresses alongside inhibitor development, not before it.

## Strong attachment: Covalent kinase inhibitors

Another important class of kinase inhibitors are covalent kinase inhibitors or type VI inhibitors (99, 100). These compounds exploit the presence of reactive amino acids in the ATP site, typically cysteine, although other residues such as lysine have also been successfully used. These cysteines are not required for catalysis, but they can be used to irreversibly block the ATP site through attachment of a small molecule. Examples used in the clinic are the EGFR inhibitors afatinib and osimertinib, used in the treatment of non–small cell lung cancer and the BTK inhibitors ibrutinib and acalabrutinib, used in the treatment of chronic lymphocytic leukemia (101, 102).

Many other protein kinases have one or more cysteine residues close to the ATP site that could potentially be targeted with a covalent warhead (103). Analysis of kinase–inhibitor complexes identified 169 different kinases that had 63 different amino acid positions close to the inhibitor, of which 17 made contact (104). This analysis is especially helpful in designing inhibitors that are selective for single members of kinase families. For example, in the NEK family of kinases, only NEK2 has a cysteine residue in the Gly-rich loop and is specifically targeted with inhibitors based on an oxindole scaffold modified with one of several types of electrophilic warhead (105). Another example is fibroblast growth factor receptor 4, the only family member to have a cysteine in its hinge region, that has also successfully been targeted with covalent inhibitors: BLU9931, armed with an acrylamide warhead (106); and roblitinib, armed with an aldehyde warhead (107). The prospects seem promising for developing highly specific covalent inhibitors against many other kinase targets, which will benefit from mature design principles and a growing number of success stories (99, 100).

## Concluding remarks

In this review, we described the common structural features that underpin protein kinase regulatory mechanisms and how they relate to kinase inhibitor discovery. The PDB currently includes the structure of over 250 human protein kinase domains, many of them in more than one conformational state, comprising a total of over 7000 molecular structures. The vast majority of the structures were derived using protein crystallography, providing high-resolution snapshots of kinase structures. However, most of these structures are of the isolated kinase domain and do not include the other domains present in the protein or other binding partners. This situation is rapidly changing with the increasing use of cryo-EM to tackle full-length kinases and their complexes, as exemplified by the progress understanding the regulation of DNA damage response kinases (reviewed by Ref. (108)). Artificial intelligence–based methods have revolutionized the business of protein structure prediction, and structural models are now available for the entire human kinome (https://alphafold.ebi.ac.uk/). Kinase biologists are only just getting started with these powerful approaches, and they currently have limited capabilities in modeling specific regulatory states or ligand-dependent conformations. Experimental approaches will continue to be important as we work toward a complete set of reliable structural models of protein kinases over the next few years. A major limitation in our knowledge of protein kinases concerns their dynamics and how this influences, and is influenced by, the binding of inhibitors. These questions have been partially addressed in a few kinases, but the field will be transformed when these approaches are applied to a broader set of kinases. It is likely that a review written 5 years from now will draw on a vastly extended dataset of kinase knowledge comprising predicted structures, experimental structures, and dynamics.

The classification of ATP-competitive kinase inhibitors is not straightforward because of the diversity of their molecular structures and the complexity of the conformational space occupied by kinase–inhibitor complexes: inhibitors may bind to more than one conformational state of the kinase; crystal structures may be misleading because the packing interactions may be the major determinant of the conformational state of a kinase. In this review, we follow the usual convention of type I, I½, II, III, and IV and use the conformational state of the kinase and the exposed pockets that the inhibitors occupy as a framework for the classification. This approach relates the binding mode of inhibitors to the molecular mechanisms of kinase regulation, which is helpful in defining which biochemical and conformational states are being targeted. A framework such as this can be expanded and adapted as we uncover inhibitors with novel binding sites and mechanisms of action. To our knowledge, the DFG-inter conformational state has not yet been targeted in any kinase, and it is not clear what form these inhibitors will take. Moreover, new allosteric sites are being explored in many kinases, and the classification of inhibitors may need to be revisited to provide clear descriptions of these binding modes and the underlying allosteric regulation mechanisms they modulate.

## Conflict of interest

The authors declare that they have no conflicts of interest with the contents of this article.
